# Analyzing political party positions through multi-language twitter text embeddings

**DOI:** 10.3389/fdata.2024.1330392

**Published:** 2024-05-30

**Authors:** Jinghui Chen, Takayuki Mizuno, Shohei Doi

**Affiliations:** ^1^Graduate Institute for Advanced Studies (SOKENDAI), Hayama, Japan; ^2^National Institute of Informatics, Tokyo, Japan; ^3^Graduate School of Law and Public Policy School, Hokkaido University, Hokkaido, Japan

**Keywords:** text analysis, multilingual NLP, LaBSE, LDA, computational social science

## Abstract

Traditional monolingual word embedding models transform words into high-dimensional vectors which represent semantics relations between words as relationships between vectors in the high-dimensional space. They serve as productive tools to interpret multifarious aspects of the social world in social science research. Building on the previous research which interprets multifaceted meanings of words by projecting them onto word-level dimensions defined by differences between antonyms, we extend the architecture of establishing word-level cultural dimensions to the sentence level and adopt a Language-agnostic BERT model (LaBSE) to detect position similarities in a multi-language environment. We assess the efficacy of our sentence-level methodology using Twitter data from US politicians, comparing it to the traditional word-level embedding model. We also adopt Latent Dirichlet Allocation (LDA) to investigate detailed topics in these tweets and interpret politicians' positions from different angles. In addition, we adopt Twitter data from Spanish politicians and visualize their positions in a multi-language space to analyze position similarities across countries. The results show that our sentence-level methodology outperform traditional word-level model. We also demonstrate that our methodology is effective dealing with fine-sorted themes from the result that political positions towards different topics vary even within the same politicians. Through verification using American and Spanish political datasets, we find that the positioning of American and Spanish politicians on our defined liberal-conservative axis aligns with social common sense, political news, and previous research. Our architecture improves the standard word-level methodology and can be considered as a useful architecture for sentence-level applications in the future.

## 1 Introduction

Theorists have commonly noted that a community's language reflects its cultural beliefs and practices (Whorf, 1956; Lévi-Strauss, 1963). These beliefs are entwined with diverse stereotypes about gender, color, occupation and so forth. Following this insight, texts have been a crucial resource for academics to delve into the exploration of cultural categories and meaning structures. However, practical research often focuses on just one or two facets due to the challenges in handling and analyzing such multidimensional data associated with diverse cultural domains (Evans and Aceves, 2016). Kozlowski et al. (2019) adopted a Word2Vec model on texts from millions of books published over 100 years and identified the ability of high-dimensional word embedding vectors for capturing complex cultural categories and interpreting widely shared cultural associations. In the Word2Vec's space, each word is represented as a vector and words are located based on “context” words that surround them in texts. This means words that frequently appear in similar contexts are placed closer to each other, while those appearing in distinct linguistic contexts are positioned further apart. Using various pairs of the vector embeddings of antonyms in a high-dimensional space, Kozlowski et al. (2019) established diverse cultural dimensions, including affluence, gender, race, and so forth. The projection of a word vector' position onto these cultural dimensions reflects its related cultural level beyond traditional simple binary or trinary classifications, allowing interpretations of this word from distinct angles even in a dense, high-dimensional language vector space. Nevertheless, a Word2Vec model can only generate vector embeddings for words in a training set, which is a 1-to-1 mapping relation, while capturing contextual meanings is difficult when facing sentence-level tasks. For large sentence-level corpora like social media data and more complex cultural dimension such as political positions, the discontinuousness in a word-level embedding space may struggle to accurately differentiate between concepts that are theoretically similar or dissimilar.

Our work aims to make a methodological contribution based on the architecture proposed by Kozlowski et al. (2019). On the one hand, we extend the standard word-level architecture to the sentence level by adopting a transformer-based language model. On the other hand, we expand the monolingual space to the multilingual space. The improved version of previous methodology allows us to compare the positions of given texts along sentence-level dimensions in a multilingual environment.

The rest of this paper is organized as follows. Section 2 elaborates prior research on cultural dimensions that were established from a monolingual word-level space to a multilingual sentence-level space. Section 3 delves into datasets and methodologies to establish the sentence-level dimension axes in a BERT space and measures projection levels of chosen objects. Section 4 describes the valuation results of our proposed methodologies on a social media dataset. Finally, Section 5 offers a comprehensive summary of this paper, followed by an explanation of its limitations and future work.

## 2 Related work

This section focuses on previous studies that proposed methods for constructing cultural dimensions. Section 2.1 explains how cultural dimensions are created based on the Word2Vec model. Section 2.2 illustrates the multilingual BERT space and cultural dimensions at the sentence level. In Section 2.3, we pivot and introduce political topics as cultural dimensions of interest, setting the stage for subsequent analysis and validation of our proposed methodologies.

### 2.1 Cultural dimensions based on Word2Vec

Word embedding models interpret textual significance in a manner similar to human comprehension (Utsumi, 2020). Various analogy types, including singular/plural relations, capital-to-country associations, and gender relationships, among others, are identifiable within the Word2Vec space (Mikolov et al., 2013). For example, the vector analogy from $\vec{cat}$ to $\vec{cats}$ mirrors that of $\vec{apple}$ to $\vec{apples}$; the vector analogy from $\vec{paris}$ to $\vec{france}$ is analogous to $\vec{tokyo}$ to $\vec{japan}$; the analogy from $\vec{man}\;to\;\vec{woman}$ parallels the relationship from $\vec{king}$ to $\vec{queen}$. These analogical properties of word embeddings enable arithmetic operations on word vectors, effectively capturing semantic relationships. Kozlowski and colleagues define cultural dimensions by taking the difference between word vectors corresponding to antonyms in a Word2Vec space. A typical example includes the creation of the affluence dimension. Since more than one pair of antonyms can be used to create an affluence dimension, by taking the arithmetic mean of several pairs of antonyms, such as $\vec{rich}$ and $\vec{poor}$ and $\vec{priceless}$ and $\vec{worthless}$, a robust affluence dimension can be established. After creating an affluence dimension, the affluence association for the sport of golf can be determined by projecting word vector $\vec{golf}$ onto the class dimension of affluence. Then a higher positive projection suggests a correlation with wealth, and more negative values imply a link to poverty. Instead of a binary classification, this method quantifies the affluence level of different sports along a determined affluence dimension. This methodology to observe widely shared understandings of individual words emphasizes that the semantic significance is encoded not just in the distance between two word vectors, but also in the direction of that distance.

Nevertheless, a significant limitation of Word2Vec cannot be neglected when dealing with words with multiple meanings. Homonyms can lead to ambiguity because the model cannot differentiate between the different senses of a word based on its usage in distinct contexts. Moreover, since each word is mapped to a single vector, Word2Vec cannot capture the nuances of language that arise from the context in which a word is used. This limits the model's ability to fully understand and represent the meaning of words in varied linguistic environments.

### 2.2 Multilingual BERT space

In recent times, transformer-based language models (LMs) have risen to prominence as the leading solutions for a multitude of natural language processing tasks. Bidirectional Encoder Representations from Transformer (BERT) is one state-of-the-art language models that uses transformer architecture to support machines to understand ambiguous language in text from learning the context of a word based on what is to the left and right of the word (Devlin et al., 2018).

For sentence-level tasks, Shen and Liu (2021) compared a pre-trained BERT model with a Word2Vec model on text classification tasks and concluded that the former outperformed the latter since it captured the contextual contents of articles (Shen and Liu, 2021). To verify our methodology on multilingual datasets, we employed a cutting-edge approach, the LaBSE model (Feng et al., 2020), developed by Google Engineers in 2020, to encode multilingual text embeddings into a shared embedding space. It extends BERT's Masked Language Model (MLM) architecture to accommodate multiple languages through a technique called Translation Language Modeling (TLM). Just like the BERT model, the LaBSE model consists of 12 layers of encoders initialized with pre-trained BERT weights. Parameters are shared among layers, and each output embedding vector has 768 dimensions. The hidden states of the classification tokens of the last layer are usually taken as sentence embeddings (Chanda, 2021).

Jha et al. (2022) adopted a pre-trained BERT space to conduct a financial sentiment social study and identified the feasibility of building a financial sentiment dimension with differences induced by both language specific positive and negative sentence embeddings. The successful projections of different finance-related sentences on the finance sentiment dimension suggests a long distance between sentence vectors representing the concept “Finance is positive” and vectors representing the concept “Finance is negative.”

We generalize the application of cultural-dimension-building architecture with sentence-level topics and multilingual contexts. Instead of the BERT model, we employ the LaBSE model to produce sentence embeddings within a multilingual high-dimensional space.

### 2.3 Political position similarities

We selected political topics to validate our methodology and primarily focused on divergence of US politicians in American politics. Unlike European countries, which often have multi-party political systems, the United States has been overwhelmingly influenced by just two political parties, Democratic Party and Republican Party over a century (Midlarsky, 1984). For many years, the simplest and most widely used method to gauge ideology in the United States has involved asking individuals to position themselves on a spectrum from “extremely conservative” to “extremely liberal.” However, survey responses have inherent weaknesses that individuals may be unwilling to accurately report their real opinions. The limitation is not necessarily mitigated by posing additional policy questions or employing more sophisticated algorithms to analyze responses (Argyle et al., 2021). The other most prevalent method for measuring ideology is the Nominate score, originating from the work of Poole and Rosenthal (2000) and their subsequent studies. These scores are derived from analyzing congressional roll-call votes, providing a quantitative assessment of lawmakers' political ideology based on their voting patterns. However, Nominate scores suffer from potential oversimplification of legislators' ideology, as they rely solely on voting records which not fully reflect the nuanced beliefs of lawmakers. Another approach is to estimate ideology based on social media datasets. Schulz et al. (2020) employed machine learning algorithms trained on manually annotated tweet samples to assess the ideological leanings of a vast number of Twitter (rebranded to X on July 23, 2023) users. This innovative approach leverages the power of machine learning to interpret and quantify ideological expressions in social media communication at a large scale. However, a significant number of tweets received more than one response along with more than one ideology label, indicating that annotators provided ideology labels even when they reported having “no idea at all.” This approach acknowledges the potential signal in these uncertain labels but also highlights a methodological challenge in ensuring the reliability and consistency of the annotated data.

We also adopted Twitter datasets to verify our proposed methodology. Twitter is one of the best social media resources for assessing the political landscape of a political party (Murthy, 2018). With its real-time updates and 280-character limit (in November 2022 when our datasets were collected) per tweet, the platform offers immediate insights into the perspectives of political figures. Nearly every US politician manages an official Twitter account, primarily as a means to communicate with the public. Our validation objective is to measure the positions of the US political actors on a liberal-conservative dimension. To establish our liberal-conservative dimension, instead of choosing contrasting sentences subjectively, we utilize the official platforms which are comprehensive documents from two parties' official websites. These two documents outline the stance of the two parties on various issues, including the economy, healthcare, education, climate change, and more. Our methodology utilizes the high-dimensional space and escapes from the potential risks brought by uncertain annotation. Moreover, current methodologies, such as Nominate scores, tend to simplify ideological positions into a binary spectrum. Our model challenges this simplification by leveraging the nuanced data available in social media, allowing for a multi-dimensional analysis of political ideologies.

To validate our methodology within a multilingual context, we chose Spain as our subject of investigation, given its notable history of pronounced political polarization. According to Edelman's 2023 report, Spain ranks among the most politically divided nations in Europe (Edelman Trust Barometer, 2023). The emergence of a far-right party, Vox, poses a challenge to the PP, which has traditionally been the cornerstone of the Spanish right. Additionally, the advent of a two-block party system has intensified the left-right divide (Vampa, 2020). This potential duopolistic structure makes the analysis and comparison with the US politics straightforward.

To measure the similarity between the political position distributions of the US and Spain, we projected the American politician embeddings and the Spanish politician user embeddings into the same LaBSE space.

## 3 Data and methodology

Section 3 describes the datasets employed and the construction of our combined methodology for measuring positions from text embeddings. Section 3.1 introduces the policy dataset applied to establish our liberal-conservative dimension. Section 3.2 explains the collection and processing of the dataset composed of tweets from political actors. Section 3.3 expounds on a procedure for extracting detailed topics from tweets employing a topic modeling method, and Section 3.4 delves into the measurement of positions in a LaBSE multilingual embedding space.

### 3.1 Policy dataset

In previous studies on determining diverse cultural dimensions, regardless of word- or sentence-level texts, both antonyms and sentences with opposite meanings were always chosen manually by the authors themselves. However, the choice of antonyms to create political dimension is quite challenging since it is beyond simple positive-negative binary situations. To obtain a comprehensive list of antonym policies to create our liberal-conservative axis, we adopted data from the official party platforms of both the Democratic and Republican parties.

The official party platform documents of the two parties serve as formal sets of principle goals which are supported by their members and form the foundation of their respective policy positions. To generally capture the policy positions of both parties, we utilized the latest platforms obtained from their official websites (Democratic National Committee, 2024; The Republican National Committee, 2024).

The objective is to identify policies that reflect opposite stances between two parties so that we can construct an abundant policy-based liberal-conservative dimension. Since creating summaries for every policy from the party platforms can be quite time consuming, we applied ChatGPT (OpenAI, 2022), a language model developed by OpenAI based on the GPT-4 architecture that one can interact with in a conversational. ChatGPT's ability to summarize text in a human-like fashion was praised by Gao et al. (2023) who concluded that it attained a high correlation with human judgments, was cost-effective, and had reproducible characteristics. We interacted with the interface developed based on ChatGPT by OpenAI and carefully designed the prompt to generate the following dataset.

*Policy dataset*: This dataset is comprised of 50 pairs of policies, each of which is presented in a separate row. Every pair of policies includes one from the Democratic Party and one from the Republican Party; the two policies reflect diametrically opposed statements.

In this policy dataset, each policy is formatted to be compatible with machine learning processes and is subject to a length constraint to ensure adequate details. The prompt used and the information generated are listed in the Supplementary material.

### 3.2 Twitter datasets

We selected two Twitter datasets to validate our methodologies. Years ago, academics started to research the position preferences of Twitter users on the social media's platform, regarding both political and non-political issues. By extracting political leaning information from Twitter texts collected during election periods, Gruzd and Roy (2014) and other academics evaluated and identified the political ideologies of politicians worldwide (Djemili et al., 2014). On Twitter's platform, politicians express distinct stances and prioritize different focus areas across a range of political issues. This situation allows for a nuanced analysis of their positions within each topic dimension.

We utilized a dataset titled “senator tweets” for the American members of Congress from the Hugging Face dataset library, created by Newhauser (Lhoest et al., 2021). This dataset is comprised of 99,693 tweets by 99 US senators in 2021. Each tweet is distinctly labeled as either Democratic or Republican. The dataset also includes labels for the screen names of the users who post the tweets. For a comparative analysis of international legislators, we focused on Spain, owing to its stark political polarization over the past decade (Paz et al., 2021). This characteristic potentially offers a pronounced political pattern relative to other European nations. We gathered tweets by Spanish politicians in 2021, filtering out individuals with fewer than 100 tweets throughout the year. The name list of Spanish politicians was extracted from the Twitter Parliamentarian Database (van Vliet, 2020). This database consists of parliamentarian names, parties and twitter ids from 26 countries, including the European Parliament. To ensure authenticity and subjectivity, retweets were excluded, retaining only original posts. These tweets were preprocessed. We converted all the text to lowercase and excised hashtags, mentions, URLs, punctuation, and non-alphanumeric characters. Following such meticulous cleansing, tweets were channeled into the pre-trained Pytorch LaBSE model (Reimers and Gurevych, 2020), accessible by a sentence transformer.

We computed each tweet embedding collected from American and Spanish politicians as well as each policy embedding generated by ChatGPT that originated from the American party platforms by feeding tweet texts into pre-trained LaBSE models. The hidden states of the classification tokens of the last layer were taken as embedding vectors. After computing all the text embedding vectors, we designed our liberal-conservative axis by taking the mean of all policy pair differences in the policy dataset.

### 3.3 Topic modeling

In addition to analyzing the general political stances of US senators, we also aim to examine their perspectives on specific issues. Given that politicians exhibit varied levels of interest in distinct topics, the content of their tweets often reflects corresponding biases. To figure out what kind of topics did the political actors post about in the senator dataset, we adopted one of the most popular topic modeling techniques, Latent Dirichlet Allocation, which provides a solid mathematical framework for topic discovery based on probability theory (Tong and Zhang, 2016). This probabilistic approach allows LDA to model the uncertainty inherent in assigning topics to documents, offering more nuanced insights into the thematic structure of the text data. Moreover, LDA does not require pre-labeled data to identify topics. This characteristic is particularly advantageous when dealing with vast or complex text corpora where manual labeling would be impractical or impossible.

### 3.4 Measuring positions in multilingual BERT space

We formulate our new sentence-level liberal-conservative dimension by taking the average of the differences between the sentence embedding vectors of contrasting policies defined in section 3.1. Equation (1) calculates the liberal-conservative dimension, where *r*_*n*_ denotes the nth conservative-side sentence and *m*_*n*_ denotes the nth liberal-side sentence. *q* represents the number of sentence pairs holding opposing statements:


(1) $$p_{x}=\frac{{\sum_{n}r}_{n}-{\sum_{m}m}_{m}}{q},$$

where *p*_*x*_ represents the liberal-conservative dimension embeddings. Then tweet projection *a*_*j*_ onto dimension *p*_*x*_ is computed as the cosine similarity between tweet j and dimension *p*_*x*_ (Equation 2):


(2) $$\begin{array}{l} a_{j}=\frac{t_{j}\cdot p_{x}}{\left|{\text{t}}_{\text{j}}||p_{x}\right|}=\frac{\Sigma_{d}t_{jd}p_{xd}}{\sqrt{\Sigma_{d}t_{jd}^{2}}\sqrt{\Sigma_{\text{d}}{\text{p}}_{\text{xd}}^{2}}}. \end{array}$$

Here *t*_*j*_ is the text embedding of tweet *j*, and *d* represents the elements of 768-dimension vectors. Therefore, *t*_*jd*_ defines the text embedding of tweet *j* on the *d*th dimension and *p*_*xd*_ represents the liberal-conservative dimension embeddings on the *d*th dimension.

## 4 Results analysis

We analyze whether the sentence-level political positions of US politicians validate our methodologies on the chosen policy dataset and Twitter datasets. Section 4.1 shows the measurement and distribution results of the positions of American politicians along liberal-conservative axis. Section 4.2 presents a comparison between the benchmark Word2Vec methodology and our proposed methodology. Section 4.3 and 4.4 explain the results of political topic extraction and topic-based political positions of US politicians based on LDA method. And Section 4.5 analyzes the projection results of Spanish politicians on the American axes to verify the multilingual characteristics of the LaBSE space.

### 4.1 Measuring the positions of American politicians

We utilized policy embeddings calculated based on the policy dataset, which consists of completely opposite views between the Democratic and Republican parties to establish our sentence-level liberal-conservative political ideology dimension. We considered Democratic embeddings “negative-side” sentences and Republican embeddings “positive-side” sentences and computed the liberal-conservative dimension with Equation (1). Then we employed the “senator tweets” dataset to verify the validity of our US liberal-conservative axis.

**Figure 1:**
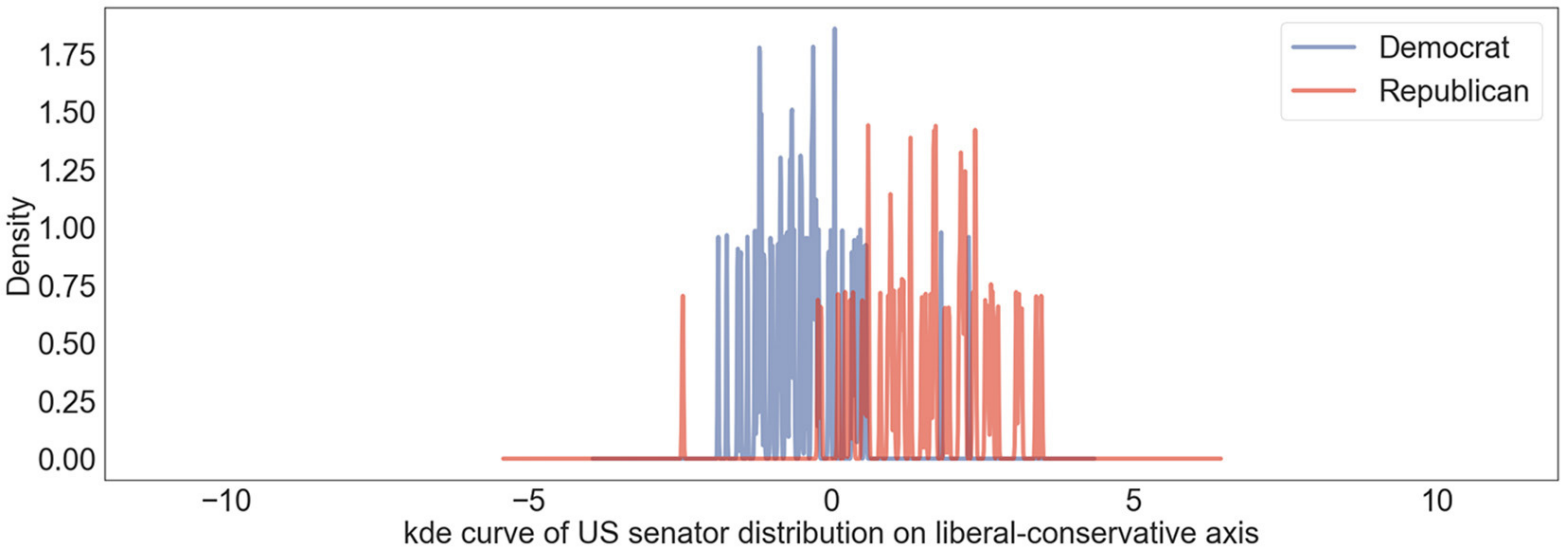
US senators' user political position distribution on US liberal-conservative axis shown in kernel density estimation curve. Blue lines represent distribution of Democratic senators and red lines denote distribution of Republican senators.

We averaged the tweet embeddings for identical users and denoted the results as American senators' embeddings. We plotted them onto the US liberal-conservative axis and visualized the projection distribution as a kernel density estimate curve (Figure 1). We normalized the distribution so that the mean value of the Democratic senators is −1 and the mean value of Republican senators is 1. In this context, users with a projection value of ~0 can be interpreted as individuals who espouse neutral stances. As observed in Figure 1, the distribution exhibits polarization, where Democratic senators are predominantly aligned with the liberal spectrum and Republican senators largely gravitate toward the conservative end.

### 4.2 A comparison with Word2Vec model

To compare the performance of the conventional Word2Vec approach with our sentence-level technique, we handpicked six antonym pairs, including “liberal/conservative” and “liberalism/conservatism,” to establish a liberal-conservative axis within Word2Vec embedding space. The list of antonym pairs is presented in the Supplementary material.

We calculated the word-level liberal-conservative dimension by averaging the differences between the selected antonym pairs and adopted Term Frequency-Inverse Document Frequency (tfIDF) to calculate weighted tweet vector embeddings. Tf-IDF is a statistical measure used to evaluate the importance of a word within a document in relation to a collection of documents (Qaiser and Ali, 2018). By calculating the weighted average of words in a tweet, we obtained the result of word-level tweet embeddings. Then politicians' positions were computed by averaging all the tweet vector projections onto word-level political dimension from the same users.

**Figure 2:**
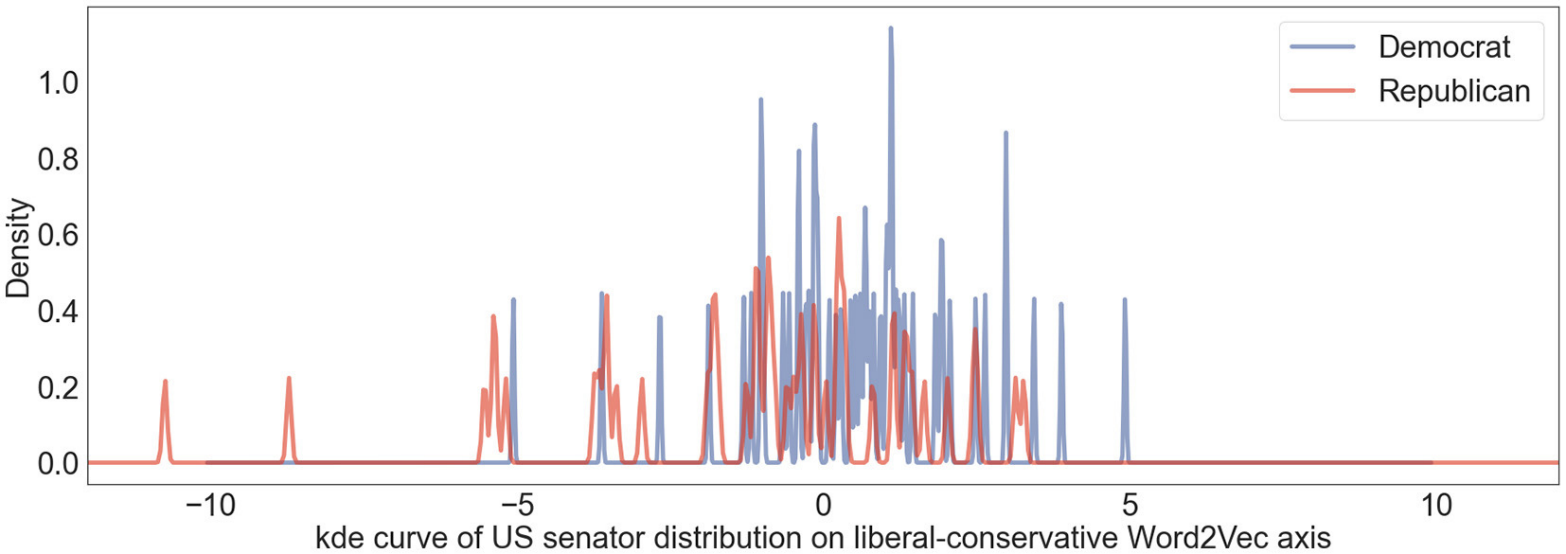
US senators' user political position distribution on US liberal-conservative Word2vec axis shown in kernel density estimation curve. Blue lines represent distribution of Democratic senators and red lines denote distribution of Republican senators.

Figure 2 shows the kernel density estimate curve of US senators' distribution on Word2Vec liberal-conservative dimension. To compare the performances of Word2Vec model and LaBSE model on the political position estimation task, we adopted the Member Ideology Data as the benchmark data which contains biographical and ideological profiles of congressional members for the selected chambers and sessions (Lewis et al., 2024). Ideological positions were derived using the DW-NOMINATE (Dynamic Weighted NOMINAL Three-step Estimation) method, a scaling procedure developed by Poole and Rosenthal in the 1980s. This approach represents legislators on a spatial map, where the proximity between two legislators indicates the similarity of their voting records. We derived the ideological positions based on the voting records of the 117th Congress (2021–2023) from the Member Ideology Data. Subsequently, we calculated the correlation metrics for both the Word2Vec model and our LaBSE model to evaluate their performance and displayed the result in Table 1. The Pearson Correlation Coefficient between the political positions on the LaBSE liberal-conservative axis and the benchmark ideology position reaches 0.73, indicating a strong correlation. The result in the Word2Vec space shows a weak correlation with a coefficient of ~0.39 with the benchmark data. According to the comparison result, our sentence-level methodology clearly outperforms the Word2Vec model.

**Table 1:** A comparison of correlation metrics between LaBSE and Word2Vec.

**Model**	**Pearson correlation coefficient (r)**	***p*-value**	**Notes**
LaBSE	0.73	<0.001	Correlation with benchmark data
Word2Vec	−0.39	<0.001	Correlation with benchmark data

### 4.3 Extracting political topics based on LDA

We extracted 100 topics from the “senator tweets” dataset based on LDA and assigned each tweet inside the dataset with a topic number from 0 to 99. We display the top 10 words for topics 0 through 5 in Table 2. The whole list of top 10 words for 100 topics is displayed in Supplementary material.

**Table 2:** Top 10 words for topics 0 through 5.

**Topic #0**	**(“broadband,” “senior,” “financial,” “result,” “dangerous,” “encourage,” “rural,” “expand,” “community,” “access”)**
Topic #1	(“virginia,” “effective,” “republican,” “concern,” “letter,” “mandate,” “democrat,” “send,” “senator,” “vaccine”)
Topic #2	(“housing,” “land,” “budget,” “priority,” “report,” “bad,” “news,” “future,” “build,” “new”)
Topic #3	(“butte,” “beth,” “grassley,” “ball,” “poison,” “inflict,” “feb,” “creek,” “outbreak,” “sight”)
Topic #4	(“disguise,” “unjustly,” “andrew,” “crackdown,” “advisory,” “torture,” “denounce,” “imprison,” “prison,” “arrest”)
Topic #5	(“need,” “spending,” “trillion,” “price,” “spend,” “cost,” “home,” “relief,” “covid,” “provide”)

We chose several topics and calculated the political positions of US senators on these topics for validation. For example, given the top 10 words: “need,” “spending,” “trillion,” “price,” “spend,” “cost,” “home,” “relief,” “covid,” “provide,” topic 5 is likely related to financial aspects of COVID-19 relief efforts. This could include discussions on the necessity and cost of relief measures, spending on various relief efforts, and financial support provided to individuals and businesses affected by the COVID-19 pandemic. Another instance is topic 8 with ten words: “women,” “vaccination,” “equal,” “black,” “food,” “team,” “win,” “deserve,” “justice,” “woman'; this suggests at topic focused on equality across different spheres, including gender, race, health, and social justice. Topic 12 includes the following words: “advocate,” “union,” “fix,” “solution,” “tell,” “agenda,” “decision,” “student,” “school,” “create'; this topic likely encompasses advocacy for educational reform or improvement, involving decision-making to enhance the educational landscape. We computed politicians' political positions respectively on these chosen topics.

### 4.4 Position similarities of politicians on distinct topics

We plotted the American senators' projection distribution on every chosen topic and analyzed the results.

Below we compared the attitudes of various US senators toward key topics in real life with their positions uncovered on our liberal-conservative axis. We gathered information sourced from the senators' official websites and news media and built our expectations on topics mentioned above: “COVID-19 relief efforts,” “equality” and “education.” After comparison, we found that these expectations align with the positions they have demonstrated on our US liberal-conservative axis.

**Figure 3:**
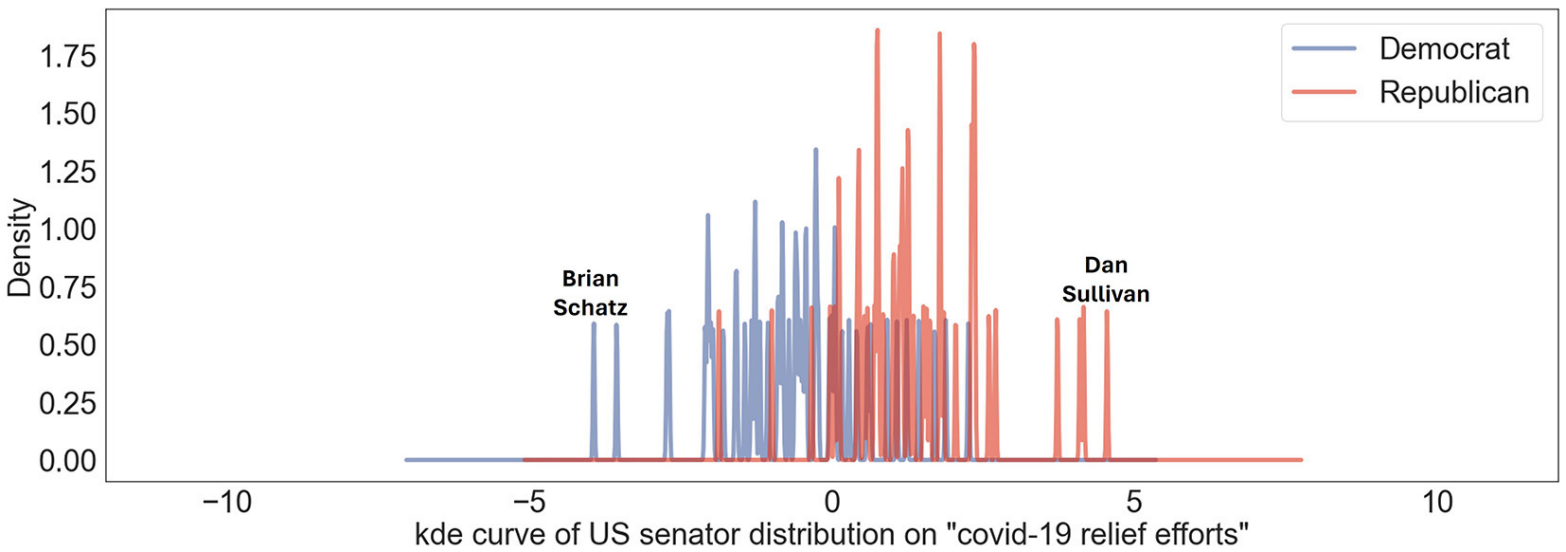
Distribution of US senators' political beliefs on US liberal-conservative axis concerning topic “COVID-19 relief efforts' is shown. Blue lines indicate viewpoints of Democratic senators; red lines depict those of Republican senators. Notable peaks associated with “Brian Schatz” and “Dan Sullivan” highlight specific stances of Senators Brian Schatz and Dan Sullivan on this spectrum.

Figure 3 shows both overlap and differentiation between Democratic and Republican senators according to the overall position of American politicians toward “COVID-19 relief efforts.” While both parties show areas of agreement, indicating shared perspectives and policies, some senators clearly align with either a distinctly liberal or conservative viewpoint regarding the financial support and relief efforts related to COVID-19. Among the senators, Brian Schatz plays a significant role in securing funding for Native Hawaiian programs through the COVID-19 relief package considered by Congress (Tsutsumi, 2021). He advocates for direct and robust federal support to manage the impact of the pandemic, highlighting the critical role of federal aid in supporting public health, economic recovery. On the other hand, the most conservative senator on our liberal-conservative axis, Dan Sullivan emphasized a multi-faceted approach to addressing the COVID-19 pandemic, including health and wellbeing, economic vitality, and fiscal stability. In 2021, Senator Sullivan publicly announced opposition to Democrats COVID relief package proposed by the Biden administration and congressional Democrats, citing a lack of bipartisan cooperation and concerns that much of the bill funds partisan policies not directly related to pandemic relief or economic recovery (Sullivan, 2021).

**Figure 4:**
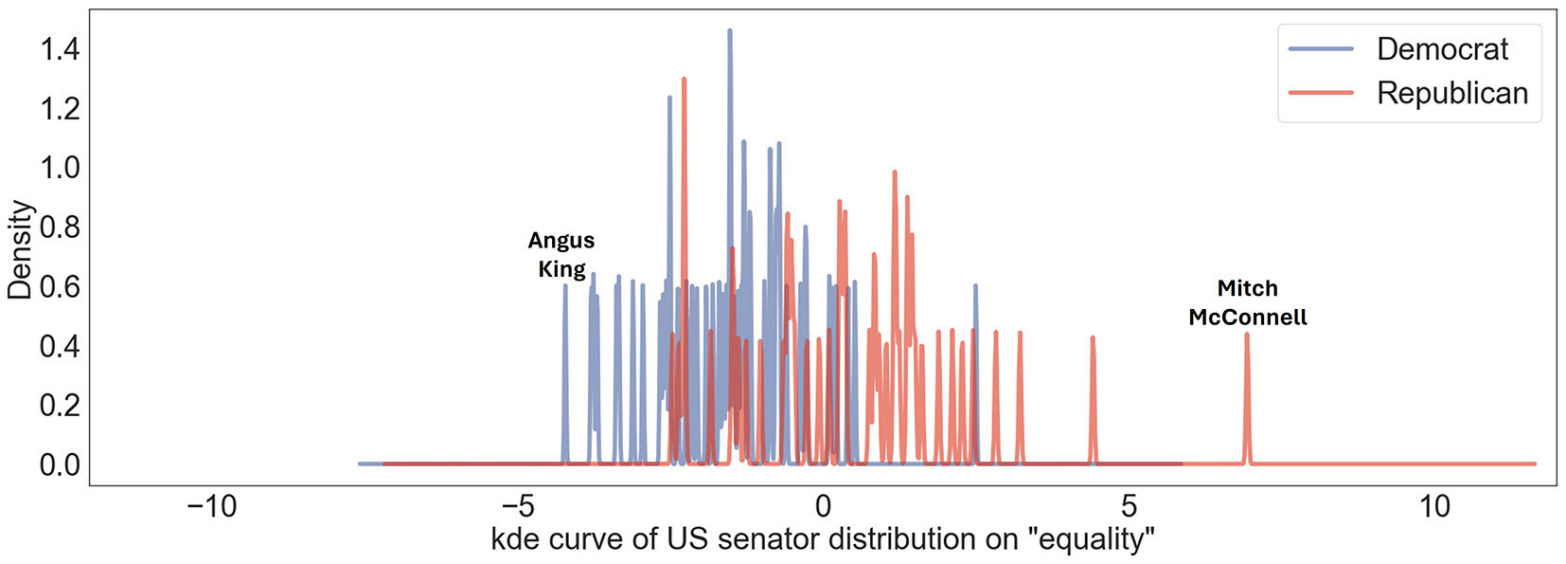
US senators' user political ideology distribution on US Liberal-Conservative axis toward topic “equality.” Democratic senators are denoted by blue lines; Republican senators are represented by red ones. Distinct peaks corresponding to “Angus King” and “Mitch McConnel” indicate particular ideological positions of Senators Angus King and Mitch McConnel on this scale.

For the topic “equality,” as presented in Figure 4, the general position among US politicians tends to lean liberal. Senator Angus King is particularly in support of LGBTQ+ rights. The Human Rights Campaign (HRC), the largest civil rights organization working to achieve LGBTQ+ equality in the United States, congratulated King upon his election, highlighting his commitment to fighting for all families, including those in the LGBTQ+ community (King, 2021). On the other hand, Leader Mitch McConnell, a Republican, has a history of opposing certain equality measures, such as The Respect for Marriage Act, which protects same-sex and interracial marriage (Mzezewa, 2022; Baska, 2024). Although he has not made public statements against interracial marriage, he has publicly opposed same-sex marriage throughout his career. He is also known for his conservative stance on LGBTQ+ rights, specifically regarding the inclusion of transgender individuals in the military (Cioffi, 2020).

**Figure 5:**
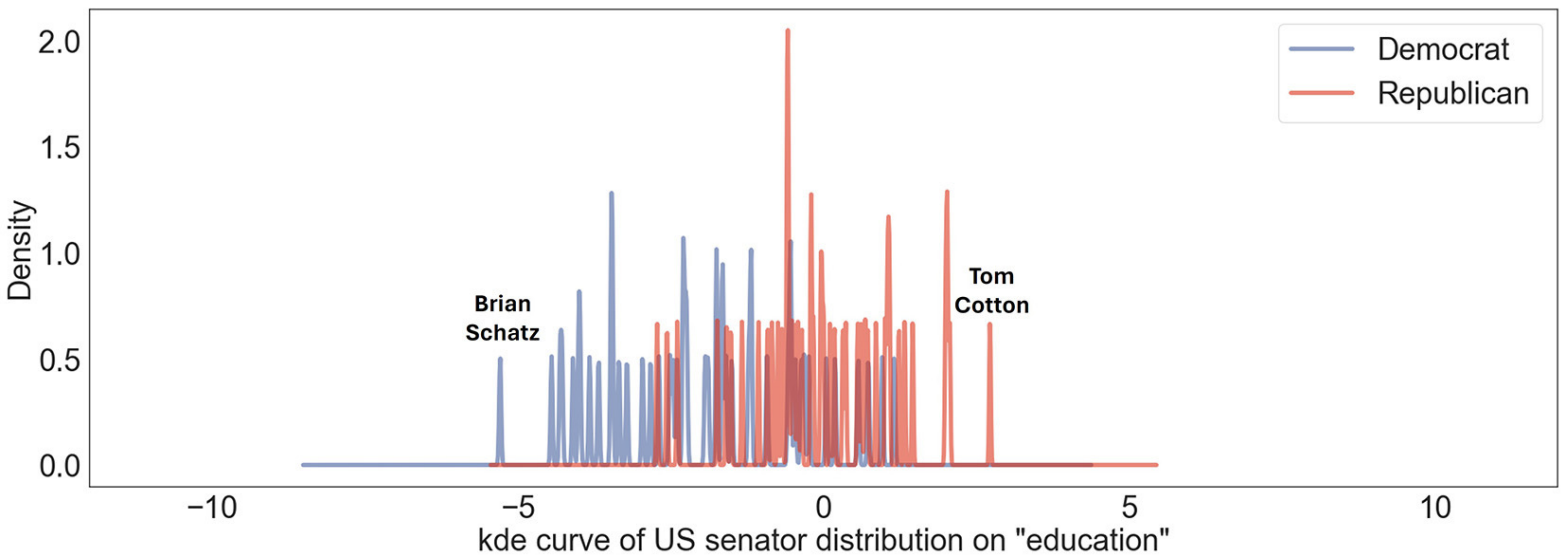
Political inclinations of US senators concerning “education” topic are mapped on US liberal-conservative ideology axis. Blue lines showcase Democratic senators' positions; red lines represent those of Republican senators. Distinct peaks corresponding to “Brian Schatz” and “Tom Cotton” indicate particular ideological positions of Senators Brian Schatz and Tom Cotton on this scale.

Regarding the topic of “education,” the prevailing sentiment among US politicians tends to lean slightly liberal. Referring to Figure 5, the most liberal senator is Brian Schatz, who has been a proactive advocate for making higher education more accessible and affordable (Kreighbaum, 2018). Nevertheless, the senator with the most conservative position, Senator Tom Cotton has concentrated on entirely distinct facets aimed at enhancing the quality of education. He has worked to ban students from using phones in schools and pushed for a bill on providing select schools with $5 million a year for the installation of lockers to store cell phones (Cheng, 2023).

These reports align with the outcomes observed in our distribution analysis.

### 4.5 Measuring Spanish politician positions

**Figure 6:**
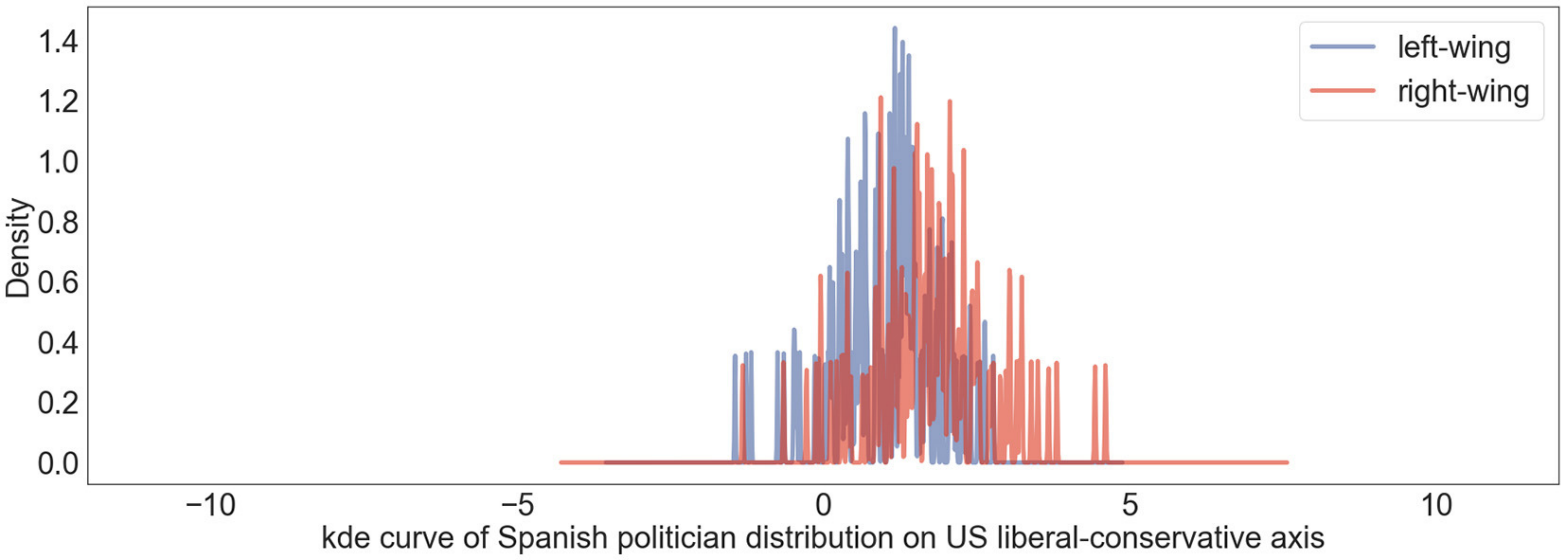
Political perspectives of Spanish politicians on US liberal-conservative spectrum are represented using a kernel density estimation curve. Blue lines denote positions of left-wing Spanish politicians; red lines highlight those of right-wing Spanish politicians.

To verify LaBSE embedding in another language, we used the Spanish politicians' Twitter dataset (described in Section 3.2) and projected their user vectors onto the US liberal-conservative axis. Such visualization helps us grasp the possible similarity between American and Spanish politics. We calculated the Spanish politician user vectors by averaging the tweet embeddings of tweets posted by the same politicians. Figure 6 shows the projection results. There is a staggering peak between the distribution of left- and right-wing users. Since in the LaBSE embedding space, contents sharing similar meanings locate close to each other regardless of the languages, we infer that the tweets from Spanish politicians show similar polarization with US politicians on the liberal-conservative dimension. Moreover, more than half of Spanish politicians' user vectors land on the conservative side of the American political ideology axis (>0), suggesting that compared to American politicians, Spanish politicians hold more conservative views. Such findings underscore the potential of our methodology for cross-cultural political discourse analysis, suggesting that despite language differences, ideological expressions retain comparable dimensions that can be captured and analyzed using LaBSE embeddings. This comparative analysis reveals a notable distribution skew toward more conservative ideologies among Spanish politicians when mapped onto the American ideological spectrum. Nonetheless, we must acknowledge the limitation arising from the lack of in-depth topic-based analysis within the Spanish Twitter dataset. We intend to conduct a more comprehensive topic-based and comparative analysis in our future research to address this gap. This section has proved the ability of basic multilingual embedding space to interpret datasets across language groups, setting the stage for more detailed analyses in future research.

## 5 Conclusions

We proposed a new architecture to establish sentence-level cultural dimensions and verified them with selected Twitter datasets. We utilized pre-trained multilingual BERT model to generate sentence embeddings and LDA methodology to extract various topics from aggregated AI-generated policy dataset. Along with verification on the American and Spanish political datasets, we conclude that the distributions of American and Spanish politicians on our defined liberal-conservative axis coincide with social common sense, political news, as well as prior research. Additionally, our methodology outperforms benchmark word-level methodology on measuring position similarities in political Twitter datasets.

Nevertheless, our proposed methodology warrants further validation across diverse datasets from different countries. Note also that our Twitter dataset is comprised of data from individuals who inherently possess selective preferences regarding topics of interest. Consequently, if a politician abstains from commenting on specific topics, the resulting user projection could be skewed. Future endeavors should enhance the representativeness and comprehensiveness of the chosen datasets.

Overall, our methodology improves the word-level methodology and help identify cultural associations among different high-dimensional cultural dimensions and even on distinct topics. Following these patterns, we offer not only an academic contribution but also a practical tool for social scientists, policymakers, and community leaders to better understand and navigate the complexities of societal beliefs and attitudes. Such insights can be invaluable in fostering mutual understanding and bridging divides in increasingly diverse societies.

## Data availability statement

The Senator Tweets dataset is publicly available and can be accessed via the following link: https://huggingface.co/datasets/m-newhauser/senator-tweets. The Spanish politicians' data was collected using the Twitter API. Queries regarding the datasets may be directed to the corresponding author.

## Ethics statement

Ethical approval was not required for the study involving human data in accordance with the local legislation and institutional requirements. The social media data was accessed and analyzed in accordance with the platform's terms of use and all relevant institutional/national regulations.

## Author contributions

JC: Writing – review & editing, Writing – original draft, Visualization, Methodology, Investigation, Data curation, Conceptualization. TM: Writing – review & editing, Supervision, Methodology, Investigation, Conceptualization. SD: Methodology, Writing – review & editing, Investigation, Conceptualization.
