# Supplementary material for: Analyzing political party positions through multi-language twitter text embeddings
Source: Front Big Data. 2024 May 30;7:1330392. doi: 10.3389/fdata.2024.1330392 (PMC11169868; doi:10.3389/fdata.2024.1330392)
Supplement: Supplementary file 1 [file Data_Sheet_1.DOCX]

Supplementary Material

# Supplementary Data

This section describes the policy dataset mentioned in section 3.1 in the main body of our paper. The dataset is generated from ChatGPT and the designed prompt to obtain the dataset and the contents generated are listed below.

## Policy dataset

Prompt: Please provide 50 nonredundant policies from 2020 Democratic platform and 2016 Republican platform respectively, where policies from Democratic platform and policies from Republican platforms hold one-to-one opposite views . Make every policy proper for machine learning.

Democratic policy list generated by ChatGPT:

1. Affirmative action should be implemented to address historical disadvantages.
2. Universal healthcare coverage should be provided for all citizens.
3. Renewable energy sources should be prioritized to combat climate change.
4. Increase taxes on the wealthy to promote income equality.
5. Stricter gun control measures should be enforced to enhance public safety.
6. Increase regulations on Wall Street to prevent financial crises.
7. Support a pathway to citizenship for undocumented immigrants.
8. Expand access to abortion services and protect reproductive rights.
9. Strengthen workers' rights and support labor unions.
10. Increase funding for public education to improve school quality.
11. Enforce stricter regulations on big corporations to prevent monopolies.
12. Expand social welfare programs to alleviate poverty.
13. Implement stricter environmental regulations to protect natural resources.
14. Advocate for stricter regulations on the pharmaceutical industry.
15. Support the legalization of marijuana and decriminalization of drug offenses.
16. Increase funding for scientific research and development.
17. Strengthen consumer protection laws to safeguard against fraud.
18. Implement stricter regulations on the use of pesticides in agriculture.
19. Support LGBTQ+ rights and work towards equal treatment.
20. Increase funding for public transportation to promote sustainable commuting.
21. Advocate for stricter regulations on campaign finance to limit corporate influence.
22. Support net neutrality to ensure equal access to the internet.
23. Invest in infrastructure development to create jobs and improve public facilities.
24. Expand access to affordable housing and combat homelessness.
25. Increase funding for mental health services and promote awareness.
26. Implement criminal justice reform to address systemic inequalities.
27. Support international agreements and alliances to promote global cooperation.
28. Protect and strengthen the Voting Rights Act to ensure fair elections.
29. Implement stricter regulations on the use of fossil fuels to combat climate change.
30. Expand access to affordable childcare and early education programs.
31. Advocate for stricter regulations on factory farming and animal welfare.
32. Support diplomatic efforts to resolve international conflicts.
33. Increase funding for arts and culture programs.
34. Strengthen regulations on workplace safety to protect employees.
35. Implement stricter regulations on the use of plastic to reduce pollution.
36. Support the rights of indigenous communities and protect their lands.
37. Expand access to affordable higher education and reduce student debt.
38. Advocate for stricter regulations on the use of genetically modified organisms (GMOs).
39. Increase funding for public parks and recreational facilities.
40. Implement stricter regulations on the use of pesticides in beekeeping.
41. Support comprehensive immigration reform to address the needs of undocumented immigrants.
42. Strengthen regulations on food labeling to promote consumer transparency.
43. Increase funding for research and treatment of diseases.
44. Advocate for stricter regulations on offshore drilling to protect marine ecosystems.
45. Expand access to affordable broadband internet in rural areas.
46. Support international efforts to combat human trafficking.
47. Increase funding for programs that promote clean energy technologies.
48. Implement stricter regulations on the use of antibiotics in livestock farming.
49. Advocate for stricter regulations on the use of artificial intelligence.
50. Strengthen regulations on the sale and possession of firearms.

Republican policy list generated by ChatGPT:

1. Reduce government regulation to promote free market competition.
2. Support a market-based healthcare system with fewer government interventions.
3. Encourage domestic energy production, including fossil fuels.
4. Lower taxes to stimulate economic growth and encourage investment.
5. Protect Second Amendment rights and oppose stricter gun control measures.
6. Reduce regulations on the financial sector to encourage innovation.
7. Secure the border and prioritize strong immigration enforcement.
8. Advocate for restrictions on abortion and protect the rights of the unborn.
9. Promote right-to-work laws and limit the power of labor unions.
10. Support school choice initiatives and reduce federal involvement in education.
11. Reduce regulations on businesses to spur economic growth.
12. Promote individual responsibility and limit the scope of social welfare programs.
13. Streamline environmental regulations to support economic development.
14. Reduce regulations on the pharmaceutical industry to encourage innovation.
15. Oppose the legalization of marijuana and enforce strict drug policies.
16. Promote private sector investments in research and development.
17. Reduce government intervention in consumer transactions and foster free markets.
18. Reduce regulations on agriculture to support farmers' autonomy.
19. Support traditional marriage and oppose policies promoting LGBTQ+ rights.
20. Promote private sector solutions for transportation infrastructure.
21. Reduce restrictions on campaign finance and promote free speech rights.
22. Oppose government interference in internet access and regulation.
23. Promote private sector investments in infrastructure projects.
24. Limit government involvement in housing and encourage market solutions.
25. Support mental health initiatives while prioritizing personal responsibility.
26. Advocate for tougher criminal sentencing and oppose criminal justice reform.
27. Prioritize American interests in international agreements and alliances.
28. Protect election integrity and oppose measures that enable voter fraud.
29. Promote domestic energy production and oppose burdensome regulations.
30. Reduce government involvement in childcare and education.
31. Oppose unnecessary regulations on agricultural practices.
32. Support a strong national defense and military presence.
33. Reduce government funding for arts and culture programs.
34. Promote voluntary workplace safety standards rather than regulations.
35. Oppose unnecessary regulations on the use of plastic.
36. Protect property rights and oppose excessive regulations on land use.
37. Reduce government involvement in higher education and promote market-based solutions.
38. Oppose unnecessary regulations on genetically modified organisms (GMOs).
39. Reduce government involvement in funding and maintaining public parks.
40. Oppose unnecessary regulations on beekeeping practices.
41. Support stricter immigration policies and border security measures.
42. Reduce government involvement in food labeling and support industry self-regulation.
43. Promote private sector investments in medical research.
44. Support domestic energy production and expand offshore drilling.
45. Encourage market-based solutions for expanding broadband access.
46. Support efforts to combat human trafficking both domestically and internationally.
47. Promote private sector investments in clean energy technologies.
48. Oppose unnecessary regulations on antibiotic use in livestock farming.
49. Promote innovation in artificial intelligence while opposing excessive regulations.
50. Protect Second Amendment rights and oppose restrictions on firearms.
